# Intracellular signaling dynamics and their role in coordinating tissue repair

**DOI:** 10.1002/wsbm.1479

**Published:** 2020-02-08

**Authors:** Samuel J. Ghilardi, Breanna M. O'Reilly, Allyson E. Sgro

**Affiliations:** ^1^ Department of Biomedical Engineering and the Biological Design Center Boston University Boston Massachusetts

**Keywords:** calcium signaling, ERK signaling, tissue repair, signaling dynamics, molecular mechanisms

## Abstract

Tissue repair is a complex process that requires effective communication and coordination between cells across multiple tissues and organ systems. Two of the initial intracellular signals that encode injury signals and initiate tissue repair responses are calcium and extracellular signal‐regulated kinase (ERK). However, calcium and ERK signaling control a variety of cellular behaviors important for injury repair including cellular motility, contractility, and proliferation, as well as the activity of several different transcription factors, making it challenging to relate specific injury signals to their respective repair programs. This knowledge gap ultimately hinders the development of new wound healing therapies that could take advantage of native cellular signaling programs to more effectively repair tissue damage. The objective of this review is to highlight the roles of calcium and ERK signaling dynamics as mechanisms that link specific injury signals to specific cellular repair programs during epithelial and stromal injury repair. We detail how the signaling networks controlling calcium and ERK can now also be dissected using classical signal processing techniques with the advent of new biosensors and optogenetic signal controllers. Finally, we advocate the importance of recognizing calcium and ERK dynamics as key links between injury detection and injury repair programs that both organize and execute a coordinated tissue repair response between cells across different tissues and organs.

This article is categorized under:

Models of Systems Properties and Processes > Mechanistic Models
Biological Mechanisms > Cell Signaling
Laboratory Methods and Technologies > Imaging
Models of Systems Properties and Processes > Organ, Tissue, and Physiological Models

## Graphical abstract

Intracellular signaling dynamics are critical for detecting injury, encoding information about an injury, and coordinating a tissue‐wide repair response. These signaling dynamics can now be spatiotemporally quantified and controlled using new technologies, linking them directly to the repair processes they coordinate.

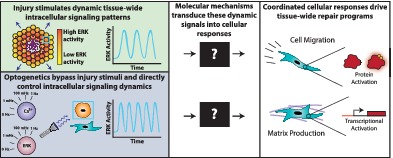

## 1 INTRODUCTION

**Figure 1:**
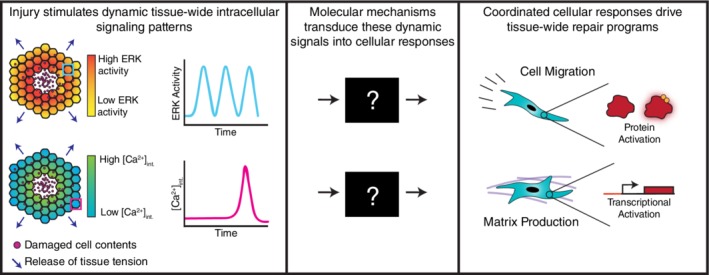
Overview of the role intracellular signaling dynamics play in tissue gap repair. (Left) Tissue injury induces both biochemical stimuli released from damaged cells and a mechanical stimulus through the release of tension. Nearby cells then sense these stimuli, which trigger the activation of intracellular signaling pathways such as calcium and ERK. These pathways can encode information by varying the concentration of a molecule or the level of activation of a protein in time and space. (Center) Molecular mechanisms inside the cell transduce dynamic calcium and ERK signals into cellular‐ and tissue‐level responses. (Right) Intracellular responses, such as protein activation and gene transcription, and tissue‐wide responses, such as cell migration and matrix production, coordinate tissue repair. ERK, extracellular signal‐regulated kinase

Wound healing is a complex process, requiring the coordination of cells from multiple tissues and organ systems across long periods of time (Bainbridge, 2013; Eming, Martin, & Tomic‐Canic, 2014; Gurtner, Werner, Barrandon, & Longaker, 2008; Shaw & Martin, 2009). Therefore, communication to coordinate cellular behaviors both within a tissue and between tissues is crucial for effective wound healing. Surprisingly, while many cellular‐level mechanisms of tissue repair have been observed (Abreu‐Blanco, Verboon, Liu, Watts, & Parkhurst, 2012; W. M. Bement, Forscher, & Mooseker, 1993; W. M. Bement, Mandato, & Kirsch, 1999; Martin & Lewis, 1992; Sakar et al., 2016; Tetley et al., 2019), little is known about how cells even in a single tissue type communicate to detect an injury and initiate repair. In a tissue repair context, it appears that injury stimulates dynamic tissue‐wide intracellular signaling patterns through extracellular stimuli such as biochemical signaling molecules or mechanical forces (Figure 1, Left). These dynamic intracellular signals are then transduced by molecular signaling mechanisms (Figure 1, Center), which in turn coordinate cellular responses that drive tissue‐wide repair programs such as coordinated cellular migration and extracellular matrix (ECM) production (Figure 1, Right; Eming et al., 2014; Hinman, Beilman, Groehler, & Sammak, 1997; Matsubayashi, Ebisuya, Honjoh, & Nishida, 2004). Observing and quantifying the cellular‐level signaling events that encode injury information and coordinate a tissue‐wide repair response is the first step toward a larger goal in wound care: active control of the injury repair process. Without a detailed understanding of how cells communicate and transduce information during injury detection and repair, we lack the information to actively correct the wound healing process when it goes awry. Until recently, the tools to make quantitative intra‐ and intercellular signaling measurements lacked either the spatial or temporal resolution needed to adequately quantify these key cellular signaling events. What classical tools have revealed are several critical intracellular signals activated after injury, the earliest being calcium and the mitogen activated protein kinase (MAPK) extracellular signal‐regulated kinase (ERK), which are essential for successful tissue repair (Handly, Pilko, & Wollman, 2015; Hinman et al., 1997; Leiper et al., 2006; Mace, 2005; Matsubayashi et al., 2004).

Given the roles of calcium and ERK as master regulators of a host of cellular signaling programs that vary both between and within organs (Clapham, 2007; Hofer & Brown, 2003; Morrison, 2012; Shaul & Seger, 2007; Wortzel & Seger, 2011), sorting out their function specifically during wound healing has been difficult. As a result, many studies have taken a reductionist approach that focuses on a single tissue layer at a time, commonly focusing on the epithelial tissue or supporting stromal tissue in an organ (Hinman et al., 1997; Matsubayashi et al., 2004; McNeil, Clarke, & Miyake, 1999; Schreier, Degen, & Baschong, 1993). Here we review the calcium and ERK signals that have been observed after injury in both epithelial and stromal tissues that are critical for detecting injury and initiating tissue repair. First, in order to understand how tissue repair occurs, we outline the known mechanisms of epithelial and stromal injury repair. Next, we detail how calcium and ERK encode injury stimuli into intracellular signaling events. In particular, we focus on the importance of spatiotemporally varying signaling dynamics and highlight the role fluorescent biosensors have played in enabling the quantification of these dynamics. Finally, we discuss the link between the information encoded by intracellular calcium and ERK signaling and potential cellular‐ and tissue‐level repair programs. This link between intracellular signaling dynamics and cellular behaviors is rapidly becoming clearer through the use of new optogenetic signal controllers that allow for direct control over intracellular signaling. Ultimately, our goal is to convey the critical roles intracellular calcium and ERK signaling dynamics play in the injury repair process, with a general aim of highlighting the importance of understanding spatiotemporally varying signal dynamics when studying cellular communication and multicellular coordination.

## 2 PHYSICAL MECHANISMS OF TISSUE REPAIR

A large portion of wound healing research has been dedicated to investigating the physical mechanisms used by cells to repair an injury, which differ between tissue types. Using both in vitro and in vivo models, several different mechanisms of wound healing have been observed. This section highlights the physical mechanisms observed during epithelial and stromal tissue repair.

### 2.1 Epithelial tissue repair

**Figure 2:**
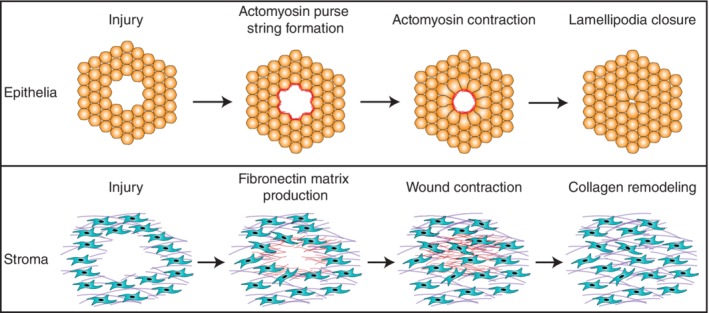
Mechanisms of epithelial and stromal injury repair. (Top) Epithelial injury repair. After injury, actin and myosin are moved to the injury edge of the epithelial cells, forming long networks of actomyosin bundles, termed the “Actomyosin Purse String” (red line). The actomyosin purse string is then contracted via the activation of myosin, which pulls the cells together and physically closes the gap. See Martin and Lewis (1992) for first image of purse string and (Tetley et al., 2019) for a recent time‐lapse video of the process. (Bottom) Stromal injury repair. After injury, fibroblasts migrate to the wound edge and secrete a provisional fibronectin matrix to fill in the gap (red lines). The fibroblasts then migrate onto the provisional matrix and continue to secrete fibronectin until the gap is closed. During the remodeling phase, the fibronectin is replaced with collagen to fully restore mechanical integrity to the tissue. See Sakar et al. (2016) for time‐lapse images of the stromal repair process

The epithelium is one of the most abundant tissue types in the body and is an essential component in the mucous membranes and glands of many organs (Marchiando, Graham, & Turner, 2010). To form this membranous structure, epithelial cells are connected by protein complexes called tight junctions to form a barrier between the external environment and internal compartments, which allows the tissue to both keep out harmful substances and retain important ones (Furuse et al., 2002). In order for a tissue wound to be fully repaired, the epithelial barrier must be fully restored through a process called re‐epithelization (Jacinto, Martinez‐Arias, & Martin, 2001). During re‐epithelization, cells at the wound margin participate in collective migration into the wound to close the tissue gap (Rousselle, Braye, & Dayan, 2018). Many modes of epithelial migration have been observed including a “leap frog” mechanism where cells roll or slide over each other (Krawczyk, 1971), lamellipodia extension (Nobes & Hall, 1999), and the contraction of a contiguous actomyosin cable called the “purse string” (Martin & Lewis, 1992; Figure 2, Top). Epithelial wound healing has been widely studied due to its experimental tractability, as it is well‐modeled in two‐dimensional tissue cultures in an assay known as a “scratch assay” in which the tissue is wounded by scratching a gap into the adhered layer of cells. In vivo, the epithelial layer is physically linked to the underlying stromal layer in many tissue types via an ECM (Yurchenco, 2011). In order for re‐epithelization to occur, the underlying ECM of the stromal tissue must first be repaired by stromal cells (Midwood, Williams, & Schwarzbauer, 2004).

### 2.2 Stromal tissue repair

Stromal tissues are the connective tissues that provide mechanical support for the epithelial tissues in an organ. Given their mechanical support function, one of the main roles of the fibroblast cells that make up the stroma is the production and remodeling of the various proteoglycans, collagens, fibronectins, laminins, and other components that compose the ECM and hold the organ together (Bainbridge, 2013; Frantz, Stewart, & Weaver, 2010). As the stroma provides mechanical support in an organ, stromal tissues are often subject to high mechanical forces (Pensalfini et al., 2018). Consequentially, the constituent stromal cells exhibit high contractility and sensitivity toward ECM‐based signaling cues (Harris, Stopak, & Wild, 1981; Lodyga et al., 2019; Pakshir et al., 2019; Tan et al., 2003). After injury, the role of stromal cells such as fibroblasts and myofibroblasts is to repair damaged ECM and close the injury site. This is generally done by secreting a provisional fibronectin matrix (Barker & Engler, 2017; R. A.F. Clark et al., 1982; Sakar et al., 2016), migrating into the wound site, and then contracting the wound closed (Bainbridge, 2013; Figure 2, Bottom). Later stages of stromal tissue repair require the production, secretion, and proper alignment of a permanent collagen matrix to replace the provisional fibronectin matrix (Welch, 1990). This dense ECM surrounding stromal cells is covered by several layers of epithelial cells, making it experimentally challenging to image stromal wound repair in vivo using conventional microscopy methods. In addition, traditional scratch assays fail to recapitulate the active mechanical environment of the stroma. Only recently has a stromal model been developed that repairs injury using a fibronectin scaffold mechanism, similar to what has been observed during in vivo stromal repair studies, opening up the opportunity for further inquiry into the molecular mechanisms controlling stromal tissue repair (R. A. F. Clark, 1998; Grinnell, Billingham, & Burgess, 1981; Legant et al., 2009; Sakar et al., 2016; To & Midwood, 2011).

## 3 INTRACELLULAR SIGNALING STIMULATED BY INJURY

The first step toward understanding the intracellular signaling responses underlying the physical mechanisms of tissue repair is to identify the specific intracellular signaling molecules and ions used by cells to detect injury. After identifying the specific intracellular signals, the next step is to identify the input signaling pathway that activates these signals, which can differ between tissue types in an organ. Two key intracellular signals activated after injury are calcium and ERK (de Roos, Willems, Peters, van Zoelen, & Theuvenet, 1997; Handly et al., 2015; Handly & Wollman, 2017; Matsubayashi et al., 2004). This section highlights the calcium and ERK signaling pathways used by cells to detect injury in epithelial and stromal tissues.

### 3.1 Calcium activation in epithelial tissue after injury

Intracellular calcium activity has long been associated with epithelial injury detection. In parallel with the discovery of the actomyosin purse string mechanism of epithelial sheet closure, several groups found that epithelial cells release intracellular calcium stores into the cytoplasm after injury in a scratch assay (K. Enomoto, Furuya, Yamagishi, & Maeno, 1992; K.‐I. Enomoto, Furuya, Yamagishi, Oka, & Maeno, 1994; Furuya, Enomoto, & Yamagishi, 1993). This result has been confirmed across different epithelia in multiple organisms (Antunes, Pereira, Cordeiro, Almeida, & Jacinto, 2013; Balaji et al., 2017; Churchill, Atkinson, & Louis, 1996; Handly et al., 2015; Handly & Wollman, 2017; Hinman et al., 1997; Sammak, Hinman, Tran, Sjaastad, & Machen, 1997; Shannon et al., 2017; Yoo, Freisinger, LeBert, & Huttenlocher, 2012). The increase in intracellular calcium is activated by the release of inositol‐1,4,5‐trisphosplate (IP_3_) from the plasma membrane, which binds to IP_3_ receptors (IP_3_R) on the endoplasmic reticulum (ER), releasing the ER's calcium stores into the cytoplasm (Mikoshiba, 2007). These calcium stores are eventually replenished by sarcoplasmic/endoplasmic reticulum calcium ATPase (SERCA) proteins by pumping cytosolic calcium back into the ER (Periasamy & Kalyanasundaram, 2007). Experiments in mammalian epithelial cell cultures have demonstrated that IP_3_ is released through cleavage of phosphatidylinositol 4,5‐bisphosphate (PI(4,5)P2) by phospholipase C at the plasma membrane. Phospholipase C is activated when adenosine triphosphate (ATP) released from damaged cells near the wound edge binds to P2y purinergic G‐coupled protein receptors (Erb & Weisman, 2012; Handly et al., 2015). More recent studies into the ATP‐calcium relationship have also indicated that calcium activation is closely linked to ERK activation after injury in epithelial tissues, of which both signals are required for re‐epithelialization (Handly et al., 2015).

### 3.2 ERK activation in epithelial tissue after injury

**Figure 3:**
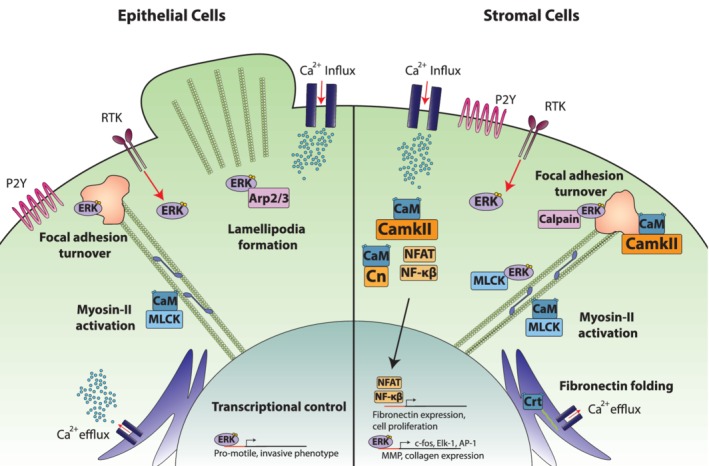
Calcium and ERK signaling networks for injury sensing and repair. Calcium and ERK can be activated by several different tissue injury signals, and can in turn activate several different tissue repair pathways. Cytosolic calcium can either come from extracellular influx via stretch activated channels, or from endoplasmic reticulum efflux. Calcium can then control several different cellular behaviors, including motility, contraction, and gene transcription. ERK can be activated by various receptor tyrosine kinases and can in turn control cellular contraction, motility, and gene transcription. One of the challenges in studying calcium and ERK signaling during injury is understanding which pathways are being activated at a given time by a given molecule, given the diverse and overlapping pathways containing calcium and ERK. Each pathway is referenced throughout the text. ERK, extracellular signal‐regulated kinase

Years of research have shown that ERK activation is not only critical for a robust epithelial response to tissue injury, but for directed cellular migration into a gap to heal a tissue wound (Aoki et al., 2017; Jiang, Zhou, Bi, & Wan, 2006; Mace, 2005; Teranishi, Kimura, & Nishida, 2009). Many tissue damage‐related factors have been identified as activators of ERK activity, including cytokines and growth factors, the aforementioned cytosolic calcium increase, and mechanical perturbations such as changes in tissue tension (Erb & Weisman, 2012; Handly & Wollman, 2017; Rosenfeldt & Grinnell, 2000; Singh, Carpenter, & Coffey, 2016). Specifically, the growth factors platelet derived growth factor, epidermal growth factor (EGF), heparin‐binding EGF‐like growth factor, and epiregulin are all potent activators of the ERK pathway (Singh et al., 2016). These growth factors first bind receptor tyrosine kinases (RTKs), and once activated, adaptor proteins and GTP exchange factors activate Ras at the cell membrane. Activated Ras then stimulates the recruitment of the Raf family of kinases to the membrane where they are activated, and in turn activate the Raf–MEK–ERK signaling pathway through a series of phosphorylation steps (Wortzel & Seger, 2011; Figure 3, left).

One major way growth factors are released into the extracellular space to serve as damage signals is through paracrine signaling systems. One paracrine damage signal is the previously discussed binding of ATP to P2y receptors on the cell membrane, which causes a rise in intracellular calcium that in turn activates matrix metallopeptidases (MMPs) to be secreted and cleave tethered EGF. This cleaved EGF acts as a paracrine signal, binding receptors on nearby cells, activating the MAPK signaling cascade, and culminating in the activation of ERK (Handly et al., 2015; L. Yang, Cranson, & Trinkaus‐Randall, 2004). Disruption of EGF cleavage using an inhibitor for the MMP ADAM17 results in an abolished ERK activation in epithelial monolayer scratch assays, confirming paracrine signaling drives the observed ERK response (Aoki et al., 2017).

Another major driver of ERK activation in epithelial cells is autocrine signaling, where EGFR binding growth factors are released as tethered ligands cleaved by the ADAM family of MMPs and bind receptors on the same cell (Sahin et al., 2004). Studies have shown that immediate proximity of autocrine signaling, rather than freely diffusing ligand, is required for inducing cell migration, again suggesting that MMPs are required for ERK activation in epithelial repair (Joslin, Opresko, Wells, Wiley, & Lauffenburger, 2007).

### 3.3 Calcium activation in stromal tissue after injury

Calcium is the primary signaling molecule triggered after stromal injury (Janssen, Mukherjee, & Ask, 2015; Lembong, Sabass, & Stone, 2017; Razzell, Evans, Martin, & Wood, 2013). Two‐dimensional scratch assays, while not perfect models of the stroma, have demonstrated that human and mouse fibroblasts release intracellular calcium in response to extracellular ATP stimuli just like epithelial cells (Lembong, Sabass, Sun, Rogers, & Stone, 2015). It is thus not unreasonable to suspect that the ATP released by damaged fibroblasts also triggers intracellular calcium release in cells in stromal tissues.

In addition to ATP, several cytokines, most notably transforming growth factor beta (TGF‐β), can also induce IP_3_‐mediated ER calcium release in fibroblasts after binding to the TGF‐β receptor in an analogous manner to ATP (McGowan et al., 2002; Mukherjee, Kolb, Duan, & Janssen, 2012; Zimmerman, Graham, Pallero, & Murphy‐Ullrich, 2013). TGF‐β is particularly important in a stromal context because it is responsible for transforming fibroblasts into myofibroblasts during wound healing by triggering the expression of smooth muscle actin (Assoian, Komoriya, Meyers, Miller, & Sporn, 1983; Desmouliere, 1993). These myofibroblasts in turn are responsible for pulling the wound closed and remodeling the provisional injury matrix into a more permanent collagen ECM (Hinz, 2016).

In addition to biochemically induced intracellular calcium store release, changes in the mechanical environment can also induce a cytosolic calcium increase in stromal cells (Kim et al., 2009; Kim et al., 2015; Munevar, Wang, & Dembo, 2004; Ruder et al., 2012), mainly through mechanically activated (also known as stretch or force activated) ion channels on the plasma membrane which open in response to mechanical forces (Coste et al., 2010; Davis, Burr, Davis, Birnbaumer, & Molkentin, 2012; Shi, Graber, Baumgart, Stone, & Cohen, 2018; Figure 3, Right). There are also reports in mesenchymal stromal cells (MSCs), the precursors of fibroblasts, that applied force can also induce ER‐mediated calcium release, independent of IP_3_ (Kim et al., 2015)_._ Given the number of potential activation pathways, calcium in stromal cells could also integrate several different stimuli during injury and activate multiple repair programs. This potential also leads to one of the current challenges we face in understanding calcium signaling's role in tissue repair: relating multiple inputs to their respective output cellular behaviors.

### 3.4 ERK activation in stromal tissue after injury

The activation of ERK in fibroblasts in some ways resembles that of epithelial cells, with the same growth factors canonically activating the MAPK signaling cascade (Kajanne et al., 2007; Yamada et al., 2018). However, it has been shown that other non‐RTK growth factor signaling pathways are also able to activate ERK in fibroblasts. Such is the case for TGF‐β, which in response to activation of its receptor, phosphorylates ShcA that then can directly activate the ERK pathway by activating SOS (Caraci et al., 2008; M. K. Lee et al., 2007; Sato, Shegogue, Hatamochi, Yamazaki, & Trojanowska, 2004). Interestingly, calcium fluxes have also been linked to the induction of ERK signaling in fibroblasts as well, with the mechanism still unknown (Lo, Luo, McCulloch, & Cruz, 1998). A combination of calcium fluxes and cytokines are required for ERK activation and subsequent gene transcription in fibroblasts. Additionally, ERK activation by cytokines has also been reported, specifically induction of ERK activation by interlukin‐4 and ‐13 (Bhogal & Bona, 2008; Lo et al., 1998).

Another potential pathway for ERK activation in stromal cells is mechanical forces. Evidence for this mechanism stems from experiments where fibroblasts are seeded in contracting collagen gels, showed that ERK is activated in response to gel contraction, and that this ERK activation controls the expression of important transcription factors (D. J. Lee, Rosenfeldt, & Grinnell, 2000; Rosenfeldt, Lee, & Grinnell, 1998). ERK activity drops in fibroblasts that are encapsulated in free floating collagen gels. Upon the reduction in ERK activity, these cells reduce their level of DNA synthesis and become quiescent. In comparison, when these same collagen gels were anchored, cells could create contractile forces, which led to high levels of ERK activity, DNA synthesis, and proliferation. Interestingly, inhibition of ERK activity impaired mechanically stimulated DNA synthesis and proliferation (Rosenfeldt & Grinnell, 2000). The mechanism by which ERK is activated under mechanical load has not yet been fully established, however it has been shown that under centrifugal force, periodontal ligament fibroblasts activate ERK through an integrin mediated RhoA‐ROCK‐FAK signaling cascade (Hong et al., 2010). These results suggest the mechanical perturbations induced by wounding may lead to ERK activation in stromal cells, yet no direct links have been made to date.

## 4 TOOLS TO MEASURE INTRACELLULAR SIGNALING DYNAMICS

The temporal or spatial resolution needed to measure dynamic signals is lacking in classical biochemical techniques such as western blotting and immunofluorescence. To overcome the limitations of these techniques, both dyes and genetically encoded biosensors have been developed that allow for real‐time measurement of intracellular signaling states. There are three main classes of fluorescent biosensors which have been developed for measuring cellular signaling dynamics: intensity‐based sensors, ratiometric sensors, and translocase sensors, each with their own benefits and drawbacks, discussed in this section.

### 4.1 Intensity‐based sensors

**Figure 4:**
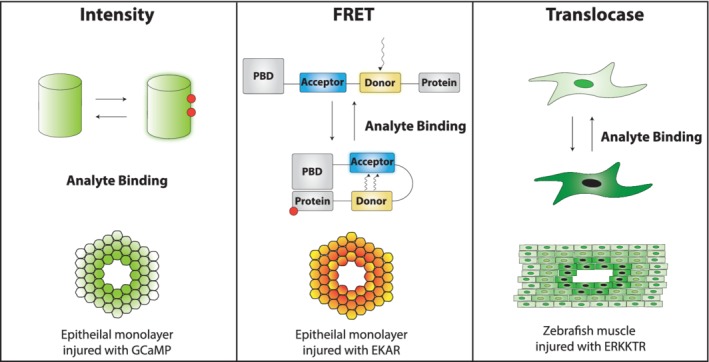
Genetically encoded biosensors for quantifying cellular signaling dynamics. (Left) Intensity‐based sensors. These sensors convert the binding of an analyte into a change in fluorescence. See Grynkiewicz, Poenie, and Tsien (1985) and Nakai, Ohkura, and Imoto (2001)) for examples of dye‐ and fluorescent protein‐based intensity sensors and (Shannon et al., 2017) for a study of calcium dynamics after epithelial injury using the GCaMP genetically encoded calcium sensor as illustrated. (Center) FRET‐based sensors. These ratiometric sensors take advantage of FRET to convert a change in distance between two fluorophores due to conformational changes in the protein into a difference in the intensity ratio between two fluorophores. As an example, in the unbound state, the donor fluorophore could be spatially separated from the acceptor fluorophore, and is not able to transfer energy, therefore there is high donor fluorescence and low acceptor fluorescence. In the bound state, the analyte binding would cause the fluorophores to move closer together, resulting in energy transfer between the two, therefore there is decreased donor fluorescence and increased acceptor fluorescence. See Heim and Tsien (1996) and Legant, Chen, and Vogel (2012) for examples of fluorescent protein and dye‐based FRET sensors in use, and Handly et al. (2015) for an application of an EKAR‐based ERK FRET sensor to quantifying epithelial wounding dynamics as illustrated. (Right) Translocase sensors. These sensors use a combination of nuclear import and export tags together with a kinase binding domain to control the nuclear translocation of a fluorescent protein. In the dephosphorylated state, the sensor resides in the nucleus. Upon kinase activation and subsequent sensor phosphorylation, the sensor is transported out of the nucleus into the cytosol. See Regot, Hughey, Bajar, Carrasco, and Covert (2014) for multiple examples of kinase translocation reporters and Mayr, Sturtzel, Stadler, Grissenberger, and Distel (2018) for an example application of the ERKKTR to understanding post‐wounding ERK dynamics in zebrafish muscle. ERK, extracellular signal‐regulated kinase

Intensity‐based sensors are among the earliest class of biosensors developed (R. Y. Tsien, Pozzan, & Rink, 1982; R. Y. Tsien, 1980), and were critical in unraveling calcium dynamics after epithelial injury. These sensors operate by converting a molecular binding event into a change in fluorescence intensity and can be made from synthetic dyes (Minta, Kao, & Tsien, 1989) as well as fluorescent proteins (Figure 4, Left; Akerboom et al., 2012; Chen et al., 2013; Dana et al., 2019; Nakai et al., 2001; Tallini et al., 2006; Tian et al., 2009). The main benefit of intensity‐based biosensors is their single fluorophore design, which allows for the multiplexing of multiple sensors (Akerboom et al., 2013), sensors and actuators (Dana et al., 2016), or sensors and other fluorescent protein fusions (Gee et al., 2014) in the limited available spectral space for fluorescent microscopy. The main drawback of intensity‐based biosensors is that it is challenging to link the sensor's intensity output to physical analyte concentrations. While protocols have been developed to calibrate sensor intensity and correlate it to analyte concentrations (Hannanta‐anan & Chow, 2016), factors such as uneven dye loading or sensor expression can hamper calibration.

### 4.2 Ratiometric sensors

Ratiometric sensors are a second class of biosensors (Grynkiewicz et al., 1985; R. Y. Tsien, 1980) and operate by converting a molecular binding event into a change in fluorescence intensity ratios of a fluorophore or pair of fluorophores. These earliest sensors are based on a single fluorophore with differential emission intensities when excited at two different wavelengths of light. Newer sensors are often designed to exploit Förster resonance energy transfer (FRET), where a change in distance modulates the energy transfer between two fluorophores, altering the ratio of fluorescence intensity of the fluorophore pair (Figure 4, Center). The majority of FRET‐based biosensor designs incorporate a fluorescent protein pair with a binding domain taken from a protein sensitive to the target signaling molecule (Heim & Tsien, 1996; Hodgson, Pertz, & Hahn, 2008; Machacek et al., 2009; Miyawaki et al., 1997), although there are hybrid variants that couple a protein binding domain to fluorescent dyes (Kraynov et al., 2000, p. 200). The main advantage of a ratiometric sensor regardless of design, is that taking the ratio of intensities internally calibrates the range of the sensor, making signal quantification much simpler by removing the dye loading or genetic expression imbalances that hinder intensity‐based sensor calibration. The main challenge when using ratiometric sensors is that single fluorophore designs often require UV light excitation which is cytotoxic (Berg, Hung, & Yellen, 2009; Minta et al., 1989), or require specialized optical equipment and filters (Ross et al., 2018). Additionally, dual fluorophore designs take up a larger part of the visible spectrum available to conventional fluorescent microscopes, limiting their ability to multiplex with other tools.

### 4.3 Translocation sensors

Translocation, or translocase, sensors are a new class of sensor only requiring a single fluorophore for signal read out. Translocase sensors works by shuttling a fluorophore between the nucleus and cytoplasm based of the activity state of the analyte. These sensors are a single polypeptide chain consisting of four distinct regions: a substrate recognition domain, a bipartite nuclear localization sequence, a nuclear escape sequence, and a fluorescent protein. Translocation sensors were designed for read out of protein kinase phosphorylation state, including ERK, Jnk, p38, and PKA, and therefore named kinase translocation reporters (KTRs; de la Cova, Townley, Regot, & Greenwald, 2017; Regot et al., 2014). Upon binding by an active, or phosphorylated kinase, the nuclear localization sequence and nuclear escape sequences are phosphorylated. This phosphorylation inhibits the nuclear localization sequence and enhances the nuclear escape sequence, so that when there is a high concentration of active kinase in the cell, the sensor will be shuttled out of the nucleus and into the cytoplasm. When there are low levels of kinase activity in the cell, the sensor is dephosphorylated by endogenous phosphatases and is shuttled back into the nucleus (Figure 4, Right). These sensors are advantageous because they only require one fluorophore for each kinase, making it easy to multiplex sensors for multiple kinases. Additionally, they are easy to engineer, as only the substrate recognition domain must be switched between sensors for different kinases (Kudo et al., 2018). It has also been suggested that KTRs have a higher dynamic range than their FRET based counterparts, and they also have faster off‐kinetics due to phosphatase accessibility (Regot et al., 2014). However, there are several downfalls of this sensor type. For example, read out is based on translocation, therefore the sensor is unable to read out spatially localized signaling dynamics, such as membrane‐localized PKA dynamics during cellular migration (Tkachenko et al., 2011). Additionally, the sensor is not reliable in cell processes where the nuclear envelope is broken down, such as during mitotic events, because the sensor read out relies on the relative concentration of fluorescence within the compartmentalized nucleus.

## 5 SPATIOTEMPORAL INTRACELLULAR SIGNALING DYNAMICS AFTER TISSUE INJURY

Using the biosensors described in Section 4, several different calcium and ERK intracellular signaling motifs have been observed. These motifs include intracellular calcium and ERK waves that spread across the tissue, as well as single cell spikes (also called flashes or pulses) of calcium and ERK activity distributed across the tissue (Aoki et al., 2017; Balaji et al., 2017; Handly et al., 2015; Handly & Wollman, 2017; Hinman et al., 1997; Hiratsuka et al., 2015; Lembong et al., 2017). Interestingly, epithelial and stromal tissues exhibit different intracellular signaling motifs, which could potentially cause the different repair behaviors observed in Section 2. Here we highlight the different calcium and ERK intracellular signaling patterns in epithelial and stromal tissues, and link them to the injury signaling pathways described in Section 3.

### 5.1 Dynamics of calcium activation after epithelial injury

In epithelia, an intracellular calcium wave propagates away from the wound immediately after tissue injury. Critically, extracellular ATP diffusion and binding to cells, and not transport from cell to cell via gap‐junction mediated IP_3_ diffusion, drives ER‐mediated calcium wave propagation, as inducing a directional flow during ATP release induces calcium activation only in the direction of flow (Handly & Wollman, 2017). However, gap junction‐mediated IP_3_ diffusion does serve to smooth out wave propagation in some epithelial systems. In Drosophila there is a distinct, smaller calcium wave that precedes the larger externally triggered injury wave. This preceding wave is local to the injury site and can potentially be induced by direct influx of extracellular calcium into damaged cells, local diffusion of IP_3_ or force activated calcium channels on the plasma membrane (Antunes et al., 2013). In either case, it requires gap‐junctions to propagate through the tissue. Gap junction‐dependent, IP_3_ induced calcium waves are also observed in the wing disk of the fly after injury (Restrepo & Basler, 2016).

While ATP is the main extracellular signal initiating calcium signaling in human epithelia, there are several other paracrine signals that can alter, but not initiate the calcium injury detection signal. In particular, several studies indicate that growth factors such as EGF and its downstream effector ERK can increase the calcium wave intensity and duration (Klepeis, Cornell‐Bell, & Trinkaus‐Randall, 2001; Takada, Furuya, & Sokabe, 2014), and in the case of some mutants, recover calcium wave behavior (Leiper et al., 2006).

### 5.2 Dynamics of ERK activation after epithelial injury

The first identification of ERK dynamics as an important component of the tissue injury response in epithelial tissues was observed in scratch assay experiments using Madin‐Darby Canine Kidney (MDCK) cells. Using immunofluorescence staining for phosphorylated ERK, distinct patterns of ERK activation were observed after wounding. Specifically, two waves of ERK activation were present; the first wave started immediately after injury and disappears within 10 min, and the second was observed to begin an hour after injury and was sustained until inactivation upon gap closure (Matsubayashi et al., 2004).

Dynamic measurements enabled by biosensors link the first of these ERK waves to the post‐wounding calcium wave in epithelial tissues. Using genetically encoded calcium and ERK sensors, Handly and colleagues observed that the initial calcium wave after injury induces a separate ERK wave across an epithelial monolayer. This ERK activation pattern has similar spatial dynamics to the previously discussed calcium wave and is activated through a paracrine signaling mechanism (Handly et al., 2015). These ERK waves have been observed both in vitro and in vivo, and moreover, this pattern also seems to be conserved across model systems ranging from mouse to zebrafish (Aoki et al., 2017; Handly et al., 2015; Hiratsuka et al., 2015; Mayr et al., 2018). Furthermore, inhibition of the initial calcium wave inhibits this ERK wave as well as later injury repair, highlighting the tight coupling between these two molecules during epithelial injury. The factors that relate these calcium and ERK dynamics after wounding are not fully understood. However, there are some soluble factors and physical cues that have been implicated in ERK activation after epithelial injury that are potential candidates to link calcium and ERK dynamics. For example, the ATP‐P2y receptor‐induced intracellular calcium wave also activates MMPs to be secreted and cleave tethered EGF. The cleaved EGF acts as a paracrine signal, binding receptors on nearby cells, activating the MAPK signaling cascade culminating in the activation of ERK (Handly et al., 2015). As mentioned previously, MMP inhibitors abolish ERK activation after wounding, abolishing the propagation of ERK waves in epithelial monolayer scratch assays (Aoki et al., 2017).

The initial calcium‐coupled ERK wave is followed by repetitive “spontaneous” ERK waves that arise behind the wound margin (Aoki et al., 2017). It is possible that the sustained activation wave seen by Matsubayashi and colleagues using immunofluorescence was due to under‐sampling, further emphasizing the need for dynamic observation of these signaling processes. Repetitive waves of ERK activation after wounding have also been observed in vivo when imaging a mouse epithelium after injury. Using transgenic mice expressing an ERK FRET biosensor in their epithelial cells, it was shown that repetitive waves of ERK activation propagate throughout the epithelial layer continuously after injury and until the closure of the tissue (Hiratsuka et al., 2015). Similar relay mechanisms have also been shown to control epithelial contractility and invagination in drosophila, where cell‐to‐cell ERK signaling through growth factor receptors creates waves with switch‐like behavior (Ogura, Wen, Sami, Shibata, & Hayashi, 2018). Another example of this wave was observed using a translocase ERK sensor in zebrafish, where it was found that both muscle and epithelial cells near a wound activate ERK in response to injury, and that this activation also spreads in a wave across the tissue, lasting about 45 min to an hour (Mayr et al., 2018). Significantly, Mayr et al. reported spontaneous secondary wound ruptures that also activated ERK signaling, noting that previous methods of measurement that require fixing and staining may have led to the misinterpretation of these events and highlighting the need for dynamic measurements.

Another potential driver of ERK dynamics in epithelial tissues is the mechanical environment. Studying ERK signaling using translocase sensors in epithelial cells seeded on varying substrate stiffness, Yang and colleagues found that ERK signaling encodes mechanosensitive information through modulating pulse frequency (J.‐M. Yang et al., 2018). This was determined by showing that optogenetic and chemical induction of protrusion increase ERK activity, and the inhibition of various membrane protrusion signaling components decreases ERK phosphorylation. Furthermore, MEK inhibition, while inhibiting ERK activity, did not abolish membrane protrusion formation, suggesting that ERK is a downstream readout of membrane protrusion (J.‐M. Yang et al., 2018), and an integrator of biochemical and mechanical signal information.

### 5.3 Dynamics of calcium activation after stromal injury

While stromal cells do share an ATP‐induced calcium wave signaling motif with epithelial cells, there are also stromal‐specific calcium signaling pathways that create unique signaling motifs in stromal cells. Unlike epithelial cells, MSCs exhibit a basal calcium spiking behavior (de Roos et al., 1997; Janssen et al., 2015; Kim et al., 2009), that can be tuned by the stiffness of the substrate the cells are plated on. Using inhibitors, it seems that MSC calcium spiking originates from ER‐mediated calcium release induced by IP_3_Rs, as well as extracellular calcium influx through both L‐type voltage‐gated and mechanically activated calcium channels. This calcium spike frequency is tuned by mechanical substrate stiffness via the activation of the Ras homolog gene family, member A GTPase (RhoA; Kim et al., 2009). In addition to this innate calcium spiking behavior, MSCs also exhibit calcium spiking when a force is applied (Kim et al., 2015). The calcium spiking induced after an applied force has a similar frequency range as basal calcium spiking. Like basal spiking, force induced spiking is mediated via a combination of ER outflux and extracellular influx of calcium, albeit the force induced spiking does not use L‐type voltage gated channels and is not IP3 mediated. In addition, basal calcium spiking does not require an intact cytoskeleton or myosin II contraction to spike while force mediated spiking requires both. Therefore, it is hypothesized that force is directly transmitted along the cytoskeleton and induces ER calcium release.

Taken together, these findings suggest that potential calcium dynamics during stromal tissue injury would have several different components. The first component would be an ATP‐induced ER mediated calcium wave, analogous to the calcium wave in epithelial cells. The other component would be an oscillatory calcium spiking signal due to the mechanical environment or applied forces. Lending credence to this idea is the observation of calcium spiking in a fibroblast monolayer after injury that was correlated with traction forces applied by cells in the monolayer (Lembong et al., 2017). The injury was created by removing a PDMS strip, so few or no cells were damaged during injury, which could explain why no calcium wave was seen.

### 5.4 Dynamics of ERK activation after stromal injury

In stromal cells, a single wave of ERK activation appears to cross the tissue layer when cells are wounded in 2D monolayer scratch assays (Matsubayashi et al., 2004; Nobes & Hall, 1999). This work was done using immunofluorescence in fixed tissues, which does not preserve dynamical information. Due to the experimental challenges presented by imaging in the stromal layer in vivo, the dynamics of ERK activation after wounding in a physiological context have not yet been studied.

## 6 SIGNALING ACTIVTAION OF CELLULAR REPAIR PROGRAMS

Once cells have detected an injury, they next have to initiate injury repair programs that coordinate cells across the tissue. In epithelia, the main goals of the repair program are cellular migration and barrier formation, and in stromal tissues the main goals of the repair program are to repair the ECM via fibronectin secretion and tissue contraction. To achieve these overall goals, cells in the tissue have to coordinate and execute many cellular processes, including migration, contraction, and changes in gene expression. Calcium and ERK have the ability to control a number of cellular behaviors in both epithelial and stromal tissues, which are summarized in this section. However, given the number of behaviors calcium and ERK can control, it has historically been difficult to identify which cellular programs are being controlled specifically for injury repair. Taking advantage of new optogenetics techniques to precisely control calcium and ERK spatiotemporal dynamics, new studies suggest that the dynamic motifs observed in tissues (such as waves and pulses) may be a key mechanism for calcium and ERK to activate specific repair response programs.

### 6.1 Control of epithelial repair by calcium signaling

The calcium wave observed immediately after injury is responsible for several post‐injury behaviors. In human epithelial monolayers, the initial calcium wave is essential for epithelial migration and wound closure (Klepeis, Weinger, Kaczmarek, & Trinkaus‐Randall, 2004). In particular, as stated above, the calcium concentration wave induces a subsequent ERK activity wave, which allows the cell to activate motility through ERK activity. (Handly et al., 2015). In drosophila, the calcium wave is synchronized with a corresponding actomyosin flow toward the wound, and disruption of the calcium wave also disrupts this actomyosin flow (Figure 3, Left). It is possible that this actomyosin and calcium flow is potentially connected to the actomyosin purse string used to close the epithelial sheet, as calcium induces myosin contraction via myosin light chain kinase (MLCK; Holzapfel, Wehland, & Weber, 1983). The calcium wave also induces an inflammatory response, via the release of H_2_O_2_. Specifically, calcium activates the enzyme DUOX, an NADPH oxidase, which increases H_2_O_2_ production and secretion, recruiting immune cells within minutes of activation (Niethammer, Grabher, Look, & Mitchison, 2009; Razzell et al., 2013; Weavers et al., 2016; Yoo et al., 2012).

### 6.2 Control of epithelial repair by ERK signaling

Given the dynamic ERK activation motifs described previously, it is likely that individual cells respond to transient, or potentially periodic, increases in ERK activity in order to response to a tissue injury. Moreover, since inhibition or knockdown of the ERK activating kinase, MEK, impairs migration of epithelial cells after wounding (Matsubayashi et al., 2004), ERK signaling is important for understanding how cells carry out tissue repair programs. ERK signaling is known to interact with cytoplasmic targets, activating other signaling pathways, components of adhesive complexes, or cytoskeletal machinery, resulting in rapid phenotypic changes. ERK signaling can also control nuclear targets such as transcription factors, thus initiating changes in gene expression and protein production on a longer timescale. Both of these ERK‐activated pathways control a number of essential tissue repair processes, such as migration, collagen secretion, and matrix degradation and contraction.

#### 6.2.1 Nontranscriptional ERK repair responses

Interrogating ERK signaling using in vivo testing of topical growth factors and agonists shows some promising results in increasing the speed of tissue repair (Uchiyama et al., 2019). However the mechanism linking ERK activation to these beneficial outcomes is not yet known. Corneal epithelial cells require ERK activation to phosphorylate the focal adhesion complex components paxillin and FAK during the formation of new focal adhesions for cell migration (Figure 3, Left), and this process is necessary for wound closure (Teranishi et al., 2009). Further, high levels of ERK activity suppress PI3K which drives membrane protrusion and lamellipodia formation at the later stages of repair (J. Li, Zhang, Soto, Woolner, & Amaya, 2013). Additionally, ERK activation is required for the activation of MMP‐2, which mediates cell contraction during migration after wounding in human lens epithelial cells (Jiang et al., 2006). It has also been suggested that ERK activation may play a role in the control or enhancement of actin polymerization in lamellipodia formation through the Arp2/3 complex (Mendoza, Vilela, Juarez, Blenis, & Danuser, 2015). Interestingly, in MCDK epithelial cells, ERK activation correlated with both a decrease in myosin light chain phosphorylation and a delayed activation (Aoki et al., 2017). It was also found that in *Xenopus* embryos, ERK is involved in the formation and contraction of an actomyosin ring around the wound. These findings raise the possibility for ERK activation to control multiple steps of the migratory response after injury.

#### 6.2.2 Transcriptional ERK repair responses

Changes in the overall transcriptional signature of epithelial cells after injury have been shown to be regulated by ERK activation, and promote a pro‐motile phenotype (Fitsialos et al., 2007). One way ERK signaling exerts transcriptional control in epithelial cells is by directly regulating ribosomal s6 kinase (RSK) phosphorylation, which then controls downstream gene expression through fos‐related antigen 1 (FRA1) mediated promoters in epithelial cells. This pathway has been shown to promote motile and invasive phenotypes and may also be implicated in the epithelial–mesenchymal transition (Doehn et al., 1993).

### 6.3 Control of stromal repair by calcium signaling

#### 6.3.1 Nontranscriptional calcium repair responses

The major nontranscriptional calcium signal responses are cellular contraction, cellular contractility, cellular motility, and ECM protein folding (Figure 3, right). In fibroblasts, cellular contraction is caused by the shortening of actomyosin bundles that make up stress fibers. These actomyosin bundles are composed of long f‐actin fibers linked with myosin II motors and are anchored to focal adhesions on the cell surface (Kreis & Birchmeier, 1980). Like myocytes, fibroblast contractions are induced by cytosolic calcium influxes. Intracellular calcium activates the adaptor protein calmodulin, which in turn binds to MLCK, which phosphorylates myosin II, initiating bundle shortening and cellular contraction. Calcium‐mediated fibroblast contraction can occur within 30 s of calcium influx (Holzapfel et al., 1983) making it one of the fastest responses to calcium signaling in the cell.

Another mechanism for calcium control of cellular contractility is by modulating the activity of RhoA, which is involved in actin polymerization, stabilization, and contraction (Lessey, Guilluy, & Burridge, 2012; Sit & Manser, 2011). When activated, RhoA binds to Rho associated kinase (ROCK), increasing cellular contractility by inactivating myosin light chain phosphatase (MLCP), the myosin inactivating counterpart to MLCK (Kawano et al., 1999). In addition, ROCK can also directly phosphorylate MLCK (Amano et al., 1996), inducing cellular contractions. Separately, ROCK can stabilize stress fiber formation by activating LIM kinase, which activated cofilin, inhibiting actin depolymerization (Maekawa et al., 1999). Since stress fibers are the primary structure responsible for fibroblast contractions, stabilizing stress fibers increases cellular contractility. Cytosolic calcium can induce RhoA activity via the activity of proline‐rich kinase‐2 (Pyk2) activity (Lev et al., 1995; Lim et al., 2008, p. 2). Pyk2 phosphorylates p190RhoGEF, which switches RhoA into an active state. Therefore, calcium spiking after stromal injury could affect cellular contractility via two routes: directly initiating cellular contractions by activating MLCK, and indirectly by activating RhoA via Pyk2 and inducing ROCK mediated MLCP inhibition and myosin phosphorylation.

Calcium can regulate focal adhesion turnover and cellular motility by activating both the phosphatase calcineurin and the kinase Ca^2+^/calmodulin‐dependent protein kinase II (CamKII), via the phosphatase calmodulin (Figure 3, right). Interestingly, a balance of kinase activity is required for cellular motility, as both constitutively active and inhibited CamKII reduces cellular motility, by either eliminating or aggregating the adaptor protein paxillin in focal adhesions. Similarly, while less is known about calcineurin, disrupting calcineurin activity disrupts the spatial gradients of integrins needed for cellular migration (Easley, Brown, Horwitz, & Tombes, 2008; Lawson & Maxfield, 1995).

Lastly, calcium can also control fibronectin and collagen I post‐translational folding. Fibronectin and collagen I both require the ER localized and calcium dependent chaperone protein calreticulin (Crt; Figure 3, right). Crt‐null mutants have impaired fibronectin and collagen I production and that overexpression of Crt increases ECM production. In addition, Crt serves as a calcium buffer in the ER, and Crt‐null mutants do not release ER calcium upon stimulation (Graham, Sweetwyne, Pallero, & Murphy‐Ullrich, 2010; Owusu, Zimmerman, & Murphy‐Ullrich, 2018; Zimmerman et al., 2013).

#### 6.3.2 Transcriptional calcium repair responses—NFAT

Calcium activates two families of transcription factors via calcineurin, the first being the nuclear factor of activated T‐cells (NFAT) and the second being the nuclear factor kappa‐light‐chain‐enhancer of activated B cells (NF‐κB; Figure 3, right). NFAT transcription factors exist in an inactive state in the cytosol and upon activation, the transcription factors translocate to the nucleus and begin gene transcription (Aramburu et al., 1999; Fisher, Yang, Medikonduri, & Jafri, 2006; Loh et al., 1996) allowing NFAT to translocate to the nucleus. The main injury repair pathways linked to NFAT in fibroblasts are related to cellular proliferation (Neal & Clipstone, 2003; Senavirathna et al., 2018) and ECM production (Cobbs & Gooch, 2007; Dooley et al., 2010; C. Li, Wang, Fu, Li, & Li, 2019; Stanisavljevic, Porta‐de‐la‐Riva, Batlle, de Herreros, & Baulida, 2011; Zimmerman et al., 2013). In regard to proliferation, a constitutive NFATc1‐mutant NIH 3T3‐L1 fibroblast line had an increased proliferation rate compared to naïve NIH 3T3 cells, and the NFATc1 mutant proliferation was insensitive to changes in serum levels, unlike naïve 3T3s (Neal & Clipstone, 2003).

The second major signaling pathway linked to NFAT activity in fibroblasts is ECM production. NFAT is linked to fibronectin production in several different studies, although the evidence is mixed on whether calcineurin/NFAT activation negatively (Ghiggeri et al., 1994; Johnson et al., 1999; Lieberman et al., 2019; Wolf, Killen, & Neilson, 1990) or positively (Davis et al., 2012; Gooch, Roberts, Cobbs, & Tumlin, 2007; Herum et al., 2013; Hirota, Ito, Miyazaki, Ebina, & Homma, 2014; Zimmerman et al., 2013) regulates ECM secretion. This discrepancy is due to several factors. First the drug Cyclosporin A (CsA) that is used to inhibit calcineurin and NFAT activity has many off‐target effects not recapitulated by other calcineurin or NFAT inhibitors (Hu et al., 2014; Nagano et al., 2006; Yamazaki et al., 2017; Zhou & Ryeom, 2014) that uniformly report a down regulation of ECM production. Second, each study focuses on the over‐production of matrix, but measures production of different matrix protein, with no clear relation to NFAT activity. Third, even accounting for drug effects, calcineurin knock‐outs generally exhibit a uniform increase in fibrosis, which cannot be abrogated by a constitutively active NFAT mutant (Irnaten et al., 2018).

Lastly, NFAT is linked to myofibroblast differentiation. Canonically, TGF‐β activates the fibroblast to myofibroblast transition, through NFAT signaling and SMAD pathway (Cobbs & Gooch, 2007; Derynck & Zhang, 2003; Herum et al., 2013; Irnaten et al., 2018) mediated production of α‐SMA. Therefore, NFAT has generally been though to play a necessary, but not sufficient role in fibroblast to myofibroblast transition. However, a recent report by TGF‐β by Kollmannsberger et al. reports TGF‐β independent (Kollmannsberger, Bidan, Dunlop, Fratzl, & Vogel, 2018) fibroblast to myofibroblast transitions, mediated by tensile forces in the tissue. Therefore, mechanically induced calcium activation of NFAT may play a role in the fibroblast to myofibroblast transition.

#### 6.3.3 Transcriptional calcium repair responses—NF‐κB

Unlike NFAT, NF‐κB has a slightly more complex activation pathway. Activation of NF‐κB is accomplished through calcium (Dolmetsch, Xu, & Lewis, 1998; Song et al., 2012; L. Zhu et al., 2011), but NF‐κB activity is increased by Protein Kinase C (PKC) activation by between 70 and 80% over calcium‐only activation (Leitges et al., 2001; Shahrestanifar, Fan, & Manning, 1999). In the extreme case, NF‐κB can be activated by the calcium‐independent PKCζ, complicating attempts to relate calcium dynamics to NF‐κB activity (Leitges et al., 2001). That being said, several studies have demonstrated across multiple cell types that NF‐κB responds to calcium dynamics in a similar manner as NFAT, making them functionally similar, even if NF‐κB has a more complex activation pathway (Dolmetsch et al., 1998; Song et al., 2012; L. Zhu et al., 2011). Similar to NFAT, NF‐κB also affects cellular proliferation. Specifically, inhibition of NF‐κB, particularly during early G1 phase, drastically inhibited cell proliferation (Guttridge, Albanese, Reuther, Pestell, & Baldwin, 1999). NF‐κB also regulates ECM production by activating fibronectin production both in mouse fibroblasts as well as hepatocytes (B.‐H. Lee, Park, Kang, Park, & Kim, 2002; Norton et al., 2004; Stanisavljevic et al., 2011; To & Midwood, 2011) and through inhibiting collagen I production by accumulating in the promoter region of collagen I to inhibit translation (Bigot et al., 2012; Grande et al., 2015; Karna, Nazaruk, Szoka, & Pałka, 2011; Figure 3, right). Together, these studies point to a potential role for calcium in activating repair programs mediated by NF‐κB to drive stromal tissue gap closure.

### 6.4 Control of stromal repair by ERK signaling

#### 6.4.1 Nontranscriptional ERK repair responses

The major nontranscriptional repair response controlled by ERK is fibroblast migration (Figure 3, right). Inhibition of ERK activation directly after wounding inhibits directional migration toward the injury (Sepe, Ferrari, Cantarella, Fioretti, & Paolella, 2013). Additionally, strong correlations between cells with increased directional persistence and higher phosphorylated ERK levels have been observed in vitro (Pan et al., 2013). One mechanism linked to this change in motility is the ability of ERK through the activation of MLCK (Klemke et al., 1997). Alternately, ERK activates the protease calpain, which is responsible for the disruption of focal adhesions allowing for the trailing edge of cells to retract during migration (Glading, Chang, Lauffenburger, & Wells, 2000). It is interesting to note that calpain is also a calcium‐dependent protease, highlighting the dual control of calcium and ERK over several key injury repair pathways. Furthermore, inhibiting ERK activity reduces both calpain activity and stabilizes focal adhesions, thus reducing cell motility and presenting further evidence ERK is key for controlling cellular motility that can lead to stromal tissue repair (Glading et al., 2000).

#### 6.4.2 Transcriptional ERK repair responses

Unlike calcium, ERK can directly activate a large number of transcription factors in fibroblasts, and therefore it can potentially control a large number of transcriptional responses during injury repair. Through these transcription factors ERK could control responses in immediate early genes, genes responsible for ECM remodeling, and fibroblast to myofibroblast transition genes. Among the immediate early genes, ERK activation most notably controls the transcription factor c‐fos (Wortzel & Seger, 2011). c‐fos along with c‐jun combine to form the activator protein‐1 (AP‐1) complex which goes on to regulate the expression of genes related to proliferation, differentiation and apoptosis (Karin, Liu, & Zandi, 1997). Other nuclear targets for ERK include the family of transcription factors Ets, including Elk‐1 which binds serum responsive elements in target gene promoters (Cruzalegui, Cano, & Treisman, 1999).

Extracellular signal‐regulated kinase can also transcriptionally regulate stromal repair by controlling the transcription of collagen for ECM remodeling in addition to regulating matrix degradation through MMPs. In response to stimulation by the cytokines IL‐4 and IL‐13, fibroblasts upregulated Type I collagen synthesis via the ERK activated transcription factor Elk‐1 (Bhogal & Bona, 2008). ERK activation by TGF‐β is essential for the upregulation of type I collagen production but through a β‐catenin dependent pathway, rather than AP‐1 mediated pathway, providing a complementary collagen activation pathway to the NF‐κβ mediated collagen inhibition pathway (Caraci et al., 2008). However, there have been conflicting reports on the impact of ERK signaling in this context, with some studies showing that lysophosphatidic acid can reduce TGF‐β stimulated Type I collagen synthesis in an ERK dependent manner (Sato et al., 2004). This suggests that ERK regulation of collagen production may be stimulus dependent and that an increase in ERK activity may not necessarily correspond to an increase in collagen production.

Collagen Type I production for stromal tissue repair can also be regulated by ERK through mechanical force. The activation of the ERK and JNK pathways in response to centrifugal force activates c‐Jun and c‐fos to form an AP‐1 transcription factor complex, which directly regulates collagen expression in human periodontal ligament fibroblasts (Kook et al., 2009). Not only is collagen production essential to tissue repair, but the remodeling of disrupted collagen in the wound site is also critical for gap closure. Specifically, ERK control of MMP production is important not only for fibroblast migration through dense ECM, but also for wound contraction. Activation of the ERK pathway through EGFR activates MMPs to degrade matrix and promotes collagen contraction by fibroblasts through the AP‐1 transcription factor complex (Kajanne et al., 2007).

Lastly, it has been shown that the transition from fibroblast to myofibroblast, which is activated in response to TGF‐β, is at least partially regulated by ERK activity. ERK activation by TGF‐β is essential for the upregulation of α‐SMA and collagen, makers of fibroblast to myofibroblast transition (Caraci et al., 2008). This myofibroblast transition highlights another potential node for signaling crosstalk between calcium and ERK, as both signals are necessary for α‐SMA production.

## 7 TOOLS TO CONTROL CELLULAR SIGNALING DYNAMICS TO ACTIVATE TISSUE REPAIR PROGRAMS

As highlighted in the preceding section, it has become clear that the dynamics of cellular signaling are important for understanding cellular communication. While fluorescent biosensors have provided the spatiotemporal resolution to observe cellular‐level signaling motifs and tissue‐wide patterns, until recently, there had not been a tool with comparable spatiotemporal resolution to control and recreate these cellular signaling dynamics. Most studies were limited to using drugs (either activators or inhibitors) or mutant models to modulate cellular signaling. While potent, both of these methods provide little to no spatial or temporal control of cellular signaling. For example, while ionophores such as ionomycin or drugs such as histamines can reliably induce a rise in intracellular calcium, it is difficult to recreate the spatial calcium waves seen in epithelia or the oscillatory calcium spikes seen in fibroblasts using these drugs. The development of light sensitive genetically encoded tools (optogenetics) now enables precise control over both the time and location of cellular signaling. Highlighted below are several optogenetic tools which could be used to explore how calcium and ERK dynamics control the tissue repair response.

### 7.1 Optogenetic calcium tools

The biggest advance in controlling intracellular calcium dynamics has come from the discovery of light controlled ion channels, which gave rise to the field of optogenetics (Tye & Deisseroth, 2012; Zhang, Wang, Boyden, & Deisseroth, 2006). By using light to stimulate the influx of ions into the cytosol, optogenetic ion channels such as channelrhodopsin‐2 (Chr2) provide both greater spatial and temporal control of intracellular cation signaling compared to drugs. Various Chr2 mutants (Kleinlogel et al., 2011; Schneider, Gradmann, & Hegemann, 2013), other opisns (Bailes & Lucas, 2013), and dimerization‐based tools such as the Cry2‐based OptoSTIM1 (Fukuda, Matsuda, & Nagai, 2014; Kyung et al., 2015; Pham, Mills, & Truong, 2011) have been developed or discovered, each with their own benefits and drawbacks. As an example of the utility of optogenetics, using a tool known as melanopsin to stimulate calcium release from the ER determined that NFAT acts by integrating calcium activity (Hannanta‐anan & Chow, 2016).

### 7.2 Optogenetic ERK tools

Control over ERK activity in cells has been enabled by various light‐inducible systems for protein dimerization, such as the protein pairs Phy and PIF, Cry2 and CIBN, and the iLID system consisting of SsrA caged by a LOV domain and SspB (Guntas et al., 2015; Kennedy et al., 2010; Levskaya, Weiner, Lim, & Voigt, 2009). For example, Phy and PIF can be induced to dimerize and dissociate using different wavelengths of light and in a system known as OptoSOS, they localize a catalytically active domain of SOS to the cell membrane and activate the ERK signaling pathway with high specificity and temporal precision (Toettcher, Weiner, & Lim, 2013). Using this tool, it was revealed that ERK activation dynamics act as a band‐pass filter for transcriptional activity of various genes. Optogenetics has also helped us understand the potential effects of the ERK activation wave seen after tissue injury. Using the Cry2‐CIBN system to control ERK dynamics and moving light in waves across a sheet of epithelial cells with a velocity that matches the naturally occurring ERK activation waves after injury, ERK‐induced collective migration occurs, creating the first causal link between ERK activation waves and directed cell migration (Aoki et al., 2017).

## 8 CONCLUSION

In this review, we have highlighted the roles that intracellular calcium and ERK signaling play in sensing tissue injury and initiating epithelial and stromal tissue repair. Both calcium and ERK integrate multiple biochemical and mechanical injury signals and initiate multiple different injury repair programs, making it difficult to connect the input injury signals to their corresponding output cellular behaviors. Here we propose that the spatiotemporal dynamics of calcium and ERK signals encode information that is the key to relating injury stimuli to downstream cellular repair programs. This section highlights three major questions for future studies to test this hypothesis.

### 8.1 How do single cells relate injury signals to repair programs?

**Figure 5:**
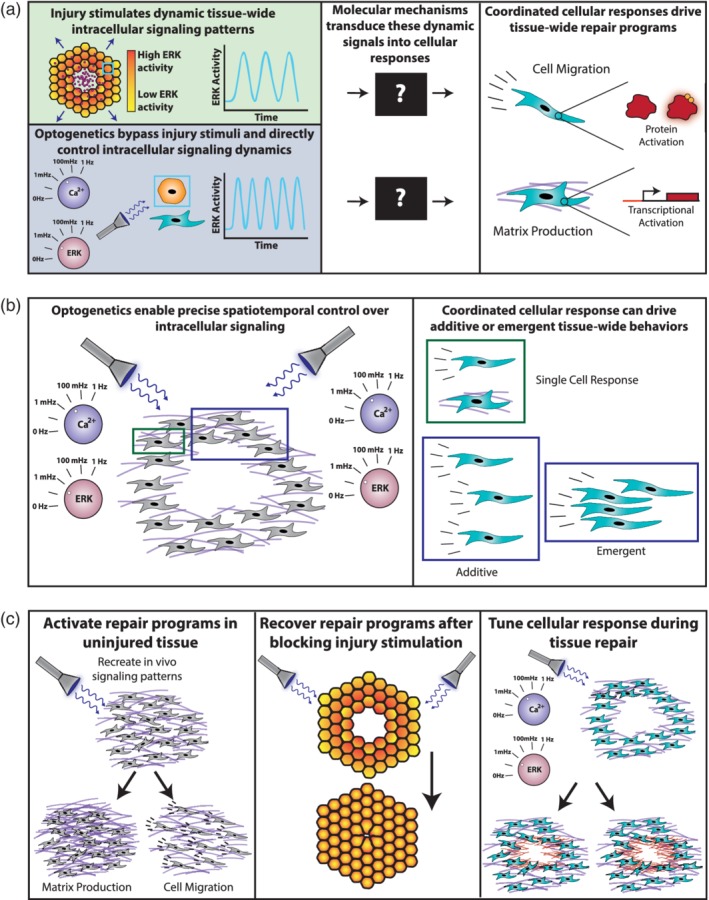
Illustration of how optogenetic controllers enable the application of classical signal processing techniques to biological systems. (a) Optogenetic controllers provide unprecedented spatiotemporal control over intracellular signals, including calcium and ERK, as illustrated by the dials tuning signal frequency. This control allows signal processing approaches such as black box techniques to be applied to the molecular processes that transduce intracellular signals into repair responses. (b) Optogenetic controllers also allow for precise spatiotemporal control over intracellular signal in a multicellular context, permitting us to determine if intracellular signaling observed across a tissue induces coordinated cellular behaviors that are merely additive of many single cells together or emergent and only exist in a group context. (c, Left) Optogenetics can be used to activate repair programs in uninjured tissues, testing the causal relationship between intracellular signaling and tissue‐wide repair programs. See Wilson et al. (2017) for an example of this technique applied to ERK activation of immediate early genes. (c, Center) Optogenetics can also test sufficiency of signaling motifs by attempting to recover tissue repair programs after blocking injury stimulation using a drug. If optogenetically induced‐intracellular signaling recovers tissue repair then the observed intracellular signaling is sufficient to control the observed tissue repair program. (c, Right) Optogenetics can also tune intracellular responses during injury repair by controlling the dynamics of intracellular signaling. This could potentially link different signaling frequencies to different levels of behavior, such as increased or decreased matrix production. ERK, extracellular signal‐regulated kinase

One of the challenges in studying cellular signaling's role in tissue repair is relating specific input injury signals to specific output injury repair behaviors. Fortunately, given the recent development of optogenetic controllers, it is now possible to perturb intact cellular signaling networks with precise spatiotemporal control and visualize the resulting intracellular calcium and ERK signaling dynamics in real‐time using fluorescent biosensors (Aoki et al., 2017; Toettcher et al., 2013; Wilson, Ravindran, Lim, & Toettcher, 2017). This allows for the application of classical signal processing techniques from electrical engineering, such as black box testing, to biological signaling networks (Figure 5a). Black box testing looks at intracellular signaling, in this case intracellular calcium or ERK signal cascade proteins (Figure 3), as an unknown quantity or “black box.” Different types of input calcium and ERK signals such as signals with different pulse frequencies are fed into the black box, and the immediate intracellular outputs such as gene transcription or protein activation, as well as later cellular behaviors such as quantity of matrix produced and cell migration speed are measured. Together, these measurements yield an input–output relationship that defines the functionality of the black box's components, or in this case, how the molecular signaling pathways discriminate between different input signals to encode different output behaviors. Different groups have applied this technique to various calcium‐ and ERK‐activated transcription factors, and have found that the transcription factors have many different temporal responses. Some act as band pass filters (Dolmetsch et al., 1998; Wilson et al., 2017), while others act as a signal integrators (Hannanta‐anan & Chow, 2016). Given the variety of injury signals outlined in Section 3 and responses outlined in Section 6, this black box approach could decipher how single epithelial and stromal cells use calcium and ERK dynamics to relate injury signals to injury repair programs.

### 8.2 How are cellular responses coordinated across stromal and epithelial tissues?

Equally important to the injury response process is how these cellular repair programs are coordinated both spatially and temporally across cells in a tissue. Tissue‐level injury responses could be predicted by combining known injury signals with the single cell input–output relationships measured in Section 8.1. However, this approach fails to account for emergent behaviors that only exist in multicellular contexts such as tissues (Figure 5b). Groups of cells can have different collective cellular signaling properties (Lembong et al., 2015; Lembong et al., 2017; Sgro et al., 2015) as well as different output cellular behaviors such as changes in cellular migration modes (Bindschadler & McGrath, 2007; Leong, Krishna, Lim, & Ladoux, 2013; B. Li & Sun, 2014; Miron‐Mendoza, Lin, Ma, Ririe, & Petroll, 2012; Sharma et al., 2015; Tambe et al., 2011). Therefore, to understand injury signaling and its role in coordinating repair responses across a tissue, both single cell injury responses and group cellular responses must be studied in a tissue‐wide context. Fortunately, the same black box techniques described in Section 8.1 can be applied to both single cells and groups of cells in a tissue. Optogenetic tools within single cells and specific groups of cells can be stimulated with high spatial control using digital micromirror devices (Arrenberg, Stainier, Baier, & Huisken, 2010; P. Zhu, Fajardo, Shum, Schärer, & Friedrich, 2012), and the resulting intracellular signals can be measured using fluorescent biosensors. Once these context‐specific cellular input–output relationships have been determined, the tissue‐wide response, including any emergent cellular behaviors, can then be understood.

### 8.3 Proof by control: Do observed intracellular signaling dynamics cause observed tissue repair behaviors?

After observing intracellular signaling and injury repair responses, it is vital to determine if the observed cellular signaling directly causes the injury repair response or if they are merely correlated. In many cases, causality can be observed by using an inhibitor drug or knock‐out mutants to block intracellular signaling, and seeing if the correlated cellular behavior still occurs. However, in the case of critical second messengers and master regulators such as calcium and ERK, simply blocking calcium and ERK signaling with a drug will inhibit a host of different cellular programs (see Sections 3 and 6; Clapham, 2007; Wortzel & Seger, 2011). Optogenetics allows for a “proof by control” approach to determining if a specific signaling motif is directly causal for a specific observed cellular behavior through using the optogenetic actuator to input a specific intracellular signal into a cell and then observing subsequent cellular behaviors. This approach could be used to activate repair programs in an uninjured tissue, analogous to the gene expression work done by Wilson et al. (2017) and the migration work done by Aoki et al. (2017; Figure 5c, Left). This technique can also be used to determine the sufficiency of the intracellular signals to activate the tissue repair behaviors observed in Section 8.2. In this experiment, optogenetics would be used to recover the previously observed intracellular signaling patterns after blocking the injury signals using a drug. If the intracellular signals are sufficient to trigger the output repair programs, then the optogenetic stimulation should result in a repaired tissue (Figure 5c, Center). If they are not sufficient, then the tissue should not be repaired. Beyond activating and recovering intracellular signals, a proof‐by‐control approach can also be used to tune tissue repair programs. Using optogenetics to control intracellular signaling (Figure 5c, Right), different repair program parameters such as cellular migration speed or quantity of matrix produced can be tuned at both the single cell and collective levels, potentially providing a pathway to not only control specific tissue repair programs, but ultimately the entire wound healing process.

### 8.4 How are tissue responses organized across a combined epithelial‐stromal organ model?

The majority of studies measuring cellular signaling have looked at a single tissue in isolation generating valuable insights into the signaling molecules and dynamics underlying injury detection and repair (de Roos et al., 1997; Handly et al., 2015; Handly & Wollman, 2017; Lembong et al., 2017; Liang, Park, & Guan, 2007; Matsubayashi et al., 2004; Sakar et al., 2016). However, coordination between epithelial and stromal tissues needs to be examined together and ultimately integrated with supporting tissues (like the cardiovascular, immune, and lymphatic systems) to model tissue communication at an organ level. A combined epithelial‐stromal model would highlight key interactions between the stroma and epithelia that cannot be recreated by single tissue models, including phenomenological interactions between stromal ECM repair and epithelial barrier formation, as well as signaling interactions based in part on diffusible signals between the epithelia and stroma. In the same way that complex tissue repair programs emerge from the coordination of cellular repair programs, there are likely novel organ level signaling motifs that emerge from coordinated tissue repair responses.

## CONFLICT OF INTEREST

The authors have declared no conflicts of interest for this article.

## AUTHOR CONTRIBTIONS

**Samuel Ghilardi:** Conceptualization; visualization; writing‐original draft; and writing‐review and editing. **Breanna O'Reilly:** Conceptualization; visualization; writing‐original draft; and writing‐review and editing. **Allyson Sgro:** Conceptualization; funding acquisition; visualization; writing‐original draft; and writing‐review and editing.

## RELATED WIREs ARTICLES

https://doi.org/10.1002/wsbm.108

https://doi.org/10.1002/wsbm.110

https://doi.org/10.1002/wsbm.1236

https://doi.org/10.1002/wsbm.1261

https://doi.org/10.1002/wsbm.16
